# Marginal Zone Lymphoma With Extensive Skeletal Involvement and Hypercalcemia: A Rare Case With a Systematic Review of the Literature

**DOI:** 10.7759/cureus.83100

**Published:** 2025-04-27

**Authors:** Soumayan Mondal, Apoorav Mahajan, Piyali Sengupta, Krushna K Sahoo, Purusottam Misra, Shubhransu Patro, Sidharth S Pattnaik

**Affiliations:** 1 Internal Medicine, Kalinga Institute of Medical Sciences, Bhubaneswar, IND; 2 Pathology, Kalinga Institute of Medical Sciences, Bhubaneswar, IND

**Keywords:** fdg pet-ct, indolent b-cell lymphoma, multifocal bone lesions, paraneoplastic manifestations, systematic literature review

## Abstract

Marginal zone lymphoma (MZL) rarely presents with skeletal involvement or hypercalcemia, making diagnosis and management particularly challenging. We report a rare case of a 44-year-old woman with extensive lytic bone lesions, hypercalcemia, and no lymphadenopathy, initially suspected to have multiple myeloma. Imaging revealed widespread osteolytic involvement, and a bone marrow biopsy confirmed MZL. She was successfully treated with rituximab-bendamustine and bisphosphonates. To contextualize this presentation, we conducted a systematic review of case reports and series describing non-Hodgkin lymphoma with skeletal disease and hypercalcemia. Across 16 studies, diffuse large B-cell lymphoma was the most common subtype, but mechanisms of hypercalcemia, including cytokine-driven osteoclast activation, parathyroid hormone-related protein (PTHrP) secretion, and vitamin D dysregulation, were consistent across subtypes. Our case illustrates the importance of considering lymphoma in patients with unexplained lytic lesions and hypercalcemia. Early biopsy, positron emission tomography-computed tomography (PET-CT) imaging, and appropriate therapy can significantly improve outcomes. This case highlights the diagnostic complexity of bone-involved lymphoma and is supported by findings from a systematic review, emphasizing the need for increased recognition of this underreported presentation.

## Introduction

Non-Hodgkin lymphoma (NHL) is a heterogeneous group of lymphoid malignancies, accounting for approximately 4% of all cancers worldwide [1]. While NHL primarily affects lymphoid tissues such as lymph nodes, spleen, and bone marrow, extranodal involvement is common, occurring in up to 30% of cases [2]. Among these extranodal sites, bone involvement is relatively rare but has significant clinical implications, including skeletal-related events, pathological fractures, and metabolic complications such as hypercalcemia [3].

Skeletal disease in NHL predominantly affects the axial skeleton, particularly the vertebrae, pelvis, and skull [4]. Unlike multiple myeloma and metastatic solid tumors, which frequently infiltrate bone, NHL-related bone lesions arise through direct tumor invasion or cytokine-driven osteoclast activation [5]. However, these lesions are often underdiagnosed, as they may be asymptomatic or present with nonspecific pain, fractures, or hypercalcemia, leading to delays in appropriate management [6].

Hypercalcemia, a well-recognized paraneoplastic complication of NHL, occurs in approximately 20% of cases, with a higher prevalence in aggressive subtypes such as diffuse large B-cell lymphoma (DLBCL) [7]. The primary mechanisms include cytokine-mediated osteoclast activation (~60%), parathyroid hormone-related protein (PTHrP) secretion (~25%), and vitamin D dysregulation (~15%), the latter being more common in T-cell lymphomas [8]. While cytokine-driven hypercalcemia predominates in NHL, PTHrP secretion is more commonly associated with solid tumors such as lung and breast cancer [9].

Diagnosing NHL-related bone disease presents unique challenges, as it frequently mimics multiple myeloma or metastatic solid tumors [10]. While multiple myeloma is distinguished by monoclonal protein (M-protein) production, NHL lesions lack this marker and can resemble osteolytic metastases. Positron emission tomography-computed tomography (PET-CT) is superior to conventional CT/magnetic resonance imaging (MRI) for detecting NHL skeletal involvement, particularly in fluorodeoxyglucose (FDG)-avid subtypes like DLBCL [2].

Despite the clinical significance of NHL bone involvement and hypercalcemia, research remains limited. Current evidence is fragmented, consisting mostly of isolated case reports and small series, making it difficult to establish clear diagnostic and therapeutic guidelines [11].

Three major gaps remain unaddressed: (1) the impact of NHL-related bone disease on prognosis and treatment response, (2) the role of bisphosphonates and denosumab in NHL skeletal disease management, and (3) the long-term consequences of NHL-related hypercalcemia on patient survival. To address this gap, we present a rare case of NHL with extensive bone involvement and severe hypercalcemia, followed by a systematic review assessing diagnostic patterns, underlying mechanisms, and treatment approaches in NHL skeletal disease.

## Case presentation

Patient presentation

A 44-year-old woman, previously in good health, presented with progressive lower back pain for one month. The pain was insidious in onset, non-radiating, and dull in character, gradually worsening and limiting her ability to perform daily activities. Two weeks prior to presentation, she developed generalized fatigue, weakness, mild nausea, and intermittent constipation. She denied fever, night sweats, weight loss, polyuria, polydipsia, fractures, nephrolithiasis, or metabolic bone disease.

Her past medical history was unremarkable, with no prior hematologic disorders, malignancies, or autoimmune conditions. She had no known family history of multiple myeloma, lymphoma, or metabolic disorders and had never smoked or consumed alcohol. There was no history of chronic steroid use, hyperparathyroidism, or renal disease.

On physical examination, she appeared fatigued but alert, with stable vital signs (blood pressure: 118/76 mmHg; heart rate: 84 bpm; respiratory rate: 16 breaths/min; oxygen saturation: 98% on room air). There was significant tenderness over the lumbar and sacral spine, but no visible deformities, joint swelling, or neurological deficits. Cardiovascular and respiratory examinations were unremarkable, and there was no palpable lymphadenopathy, hepatosplenomegaly, or abdominal masses.

Investigations

The initial biochemical workup revealed marked hypercalcemia, with a corrected serum calcium level of 13.9 mg/dL and ionized calcium of 1.61 mmol/L (Table 1). Parathyroid hormone (PTH) was suppressed (<10 pg/mL), ruling out primary hyperparathyroidism. Further workup showed normal PTH-related peptide (PTHrP) levels (1.2 pmol/L) and normal 1,25-dihydroxyvitamin D levels, suggesting that humoral hypercalcemia and vitamin D dysregulation were unlikely causes. Electrocardiography revealed a normal sinus rhythm with a shortened corrected QT interval (QTc 340 ms) as per Bazett's formula, consistent with hypercalcemia.

**Table 1:** Laboratory investigations at presentation This table summarizes the laboratory and hematologic findings of the patient at presentation. PTH: parathyroid hormone; PTHrP: parathyroid hormone-related protein; LDH: lactate dehydrogenase; SPEP: serum protein electrophoresis

Parameter	Result	Reference range
Serum calcium (mg/dL)	13.9	8.6-10.3
Ionized calcium (mmol/L)	1.61	1.15-1.33
Serum creatinine (mg/dL)	1.2	0.6-1.3
PTH (pg/mL)	<10	15-65
PTHrP (pmol/L)	1.2	<2.0
1,25-Dihydroxyvitamin D (pg/mL)	Normal	-
Alkaline phosphatase (U/L)	98	44-147
Hemoglobin (g/dL)	9.1	12-16
White blood cell count (/mm³)	4,500	4,000-11,000
Platelets (/mm³)	175,000	150,000-450,000
LDH (U/L)	540	<250
Beta-2 microglobulin (mg/L)	3.9	<3.0
SPEP	No M-spike	-
Serum free light chain ratio	1.07	0.26-1.65
Urine Bence-Jones protein	Negative	-

Given the presence of severe hypercalcemia without a clear endocrine cause, additional hematologic evaluation was pursued to rule out multiple myeloma, lymphoma, and metastatic carcinoma. Serum protein electrophoresis (SPEP) did not reveal an M-spike, and serum free light chain (FLC) analysis demonstrated a normal kappa-to-lambda ratio. Urine Bence-Jones protein was negative. A complete blood count (CBC) showed mild anemia (hemoglobin: 9.1 g/dL), normal white blood cell (WBC) count, and normal platelets. Serum lactate dehydrogenase (LDH) was elevated (540 U/L), and beta-2 microglobulin was mildly increased (3.9 mg/L) (Table 1). These findings raised suspicion for a lymphoproliferative disorder, prompting further imaging.

**Figure 1:**
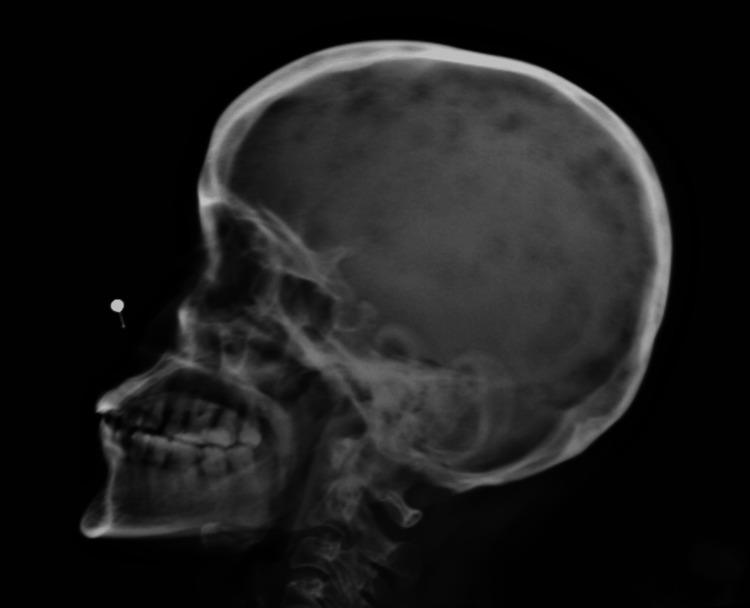
Lateral skull X-ray demonstrating multiple lytic lesions in marginal zone lymphoma Lateral skull radiograph of the present case shows multiple well-defined, punched-out lytic lesions without surrounding sclerosis, indicative of extensive osteolytic bone involvement. The absence of periosteal reaction or reactive bone formation suggests a hematologic malignancy-driven osteolysis.

**Figure 2:**
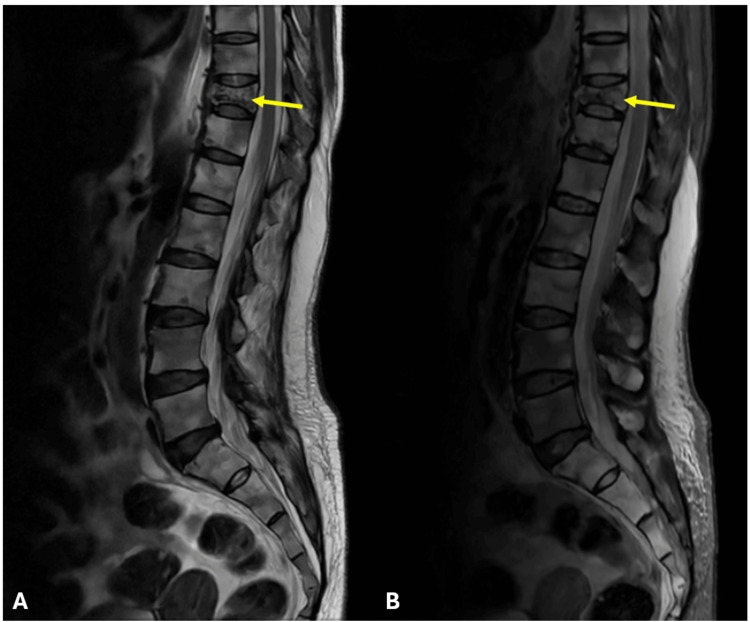
MRI of the spine (sagittal T2 and STIR) demonstrating D10 compression fracture in marginal zone lymphoma Sagittal MRI of the thoracolumbar spine showing (A) T2-weighted image and (B) STIR sequence in a patient with marginal zone lymphoma. The yellow arrow indicates a D10 vertebral compression fracture with marrow infiltration, seen as hypointensity on T2 and hyperintensity on STIR, consistent with lymphoma-associated skeletal involvement. MRI: magnetic resonance imaging; STIR: short tau inversion recovery

A lateral skull X-ray revealed multiple well-defined punched-out lytic lesions, and a skeletal survey showed similar discrete lytic lesions in the pelvis, femur, and vertebral bodies (Figure 1). MRI of the spine demonstrated diffuse marrow infiltration of vertebrae with T1 hypointensity and short tau inversion recovery (STIR) hyperintensity, along with a D10 vertebral compression fracture (50% height loss) without spinal cord compression (Figure 2).

**Figure 3:**
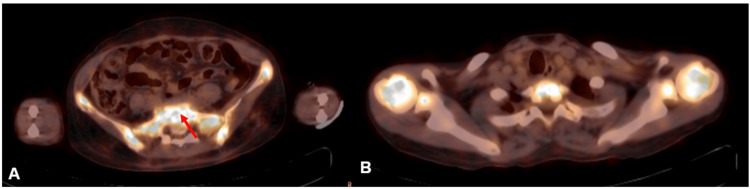
18F-FDG PET-CT showing hypermetabolic sacral involvement in marginal zone lymphoma Axial PET-CT scans showing (A) intense FDG uptake (SUVmax=19.5) in the sacrum (red arrow), consistent with lymphoma-associated skeletal involvement, and (B) heterogeneous FDG uptake in the bilateral humerus and vertebrae, indicating multifocal bone disease. PET-CT: positron emission tomography-computed tomography; FDG: fluorodeoxyglucose; SUVmax: maximum standardized uptake value

A contrast-enhanced CT (CECT) of the chest, abdomen, and pelvis was performed, revealing multiple lytic lesions in the iliac bones, sacrum, and proximal femur without significant lymphadenopathy or visceral organ involvement. A whole-body 18F-FDG PET-CT scan demonstrated FDG-avid lytic lesions with a maximum standardized uptake value (SUVmax) of 19.5 in the sacrum, but notably, no hypermetabolic lymphadenopathy or visceral organ involvement (Figure 3).

**Figure 4:**
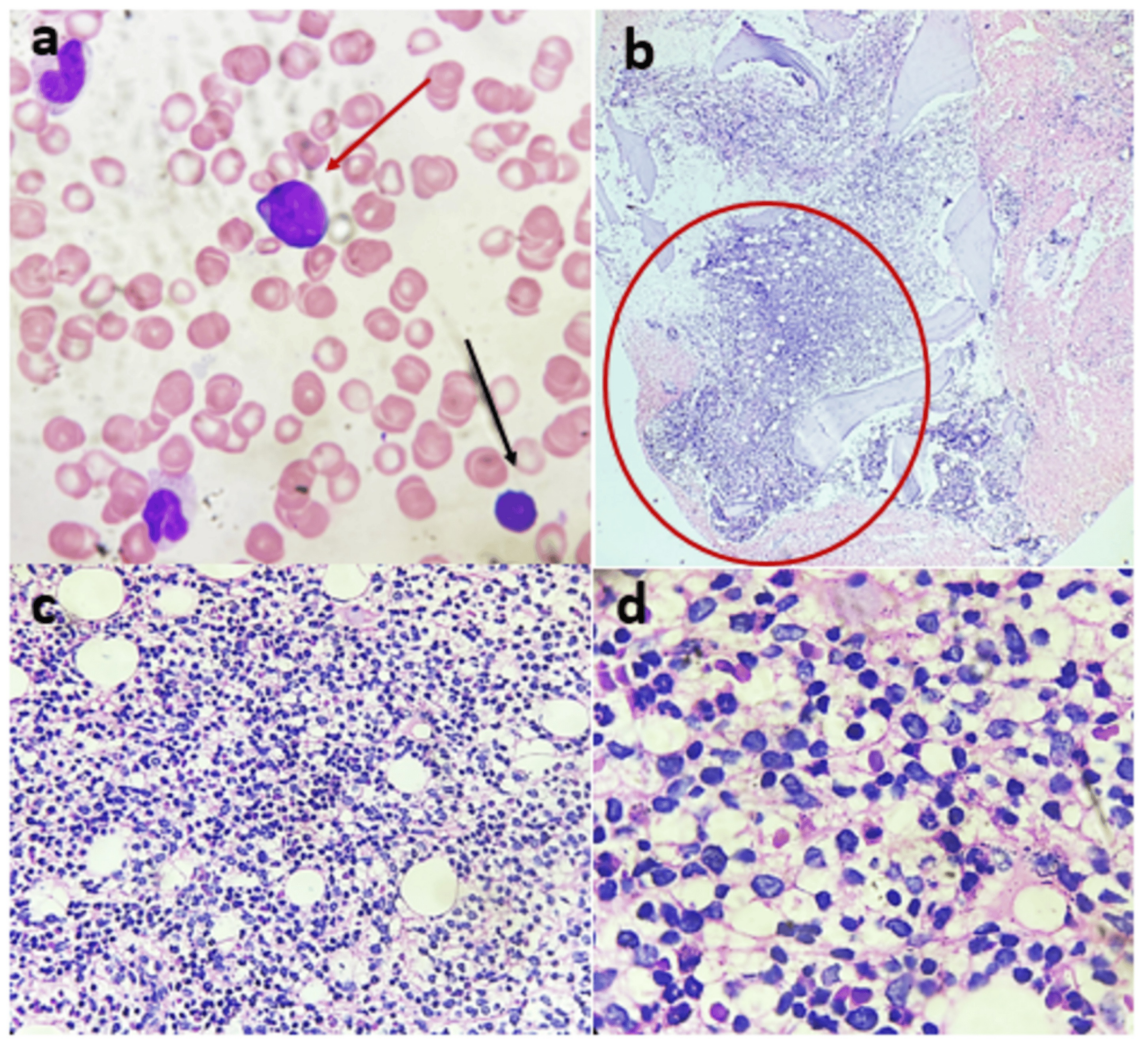
Bone marrow aspirate and biopsy findings (a) Bone marrow aspirate showing scattered suspicious lymphoid cells (red arrow) and a normal lymphocyte (black arrow). (b) Bone marrow biopsy demonstrating interstitial aggregates of small-to-intermediate-sized lymphoid cells (H&E, 200×). (c) Higher magnification of interstitial lymphoid infiltration (H&E, 400×). (d) Oil immersion view highlighting dispersed lymphoid aggregates. H&E: hematoxylin and eosin

**Figure 5:**
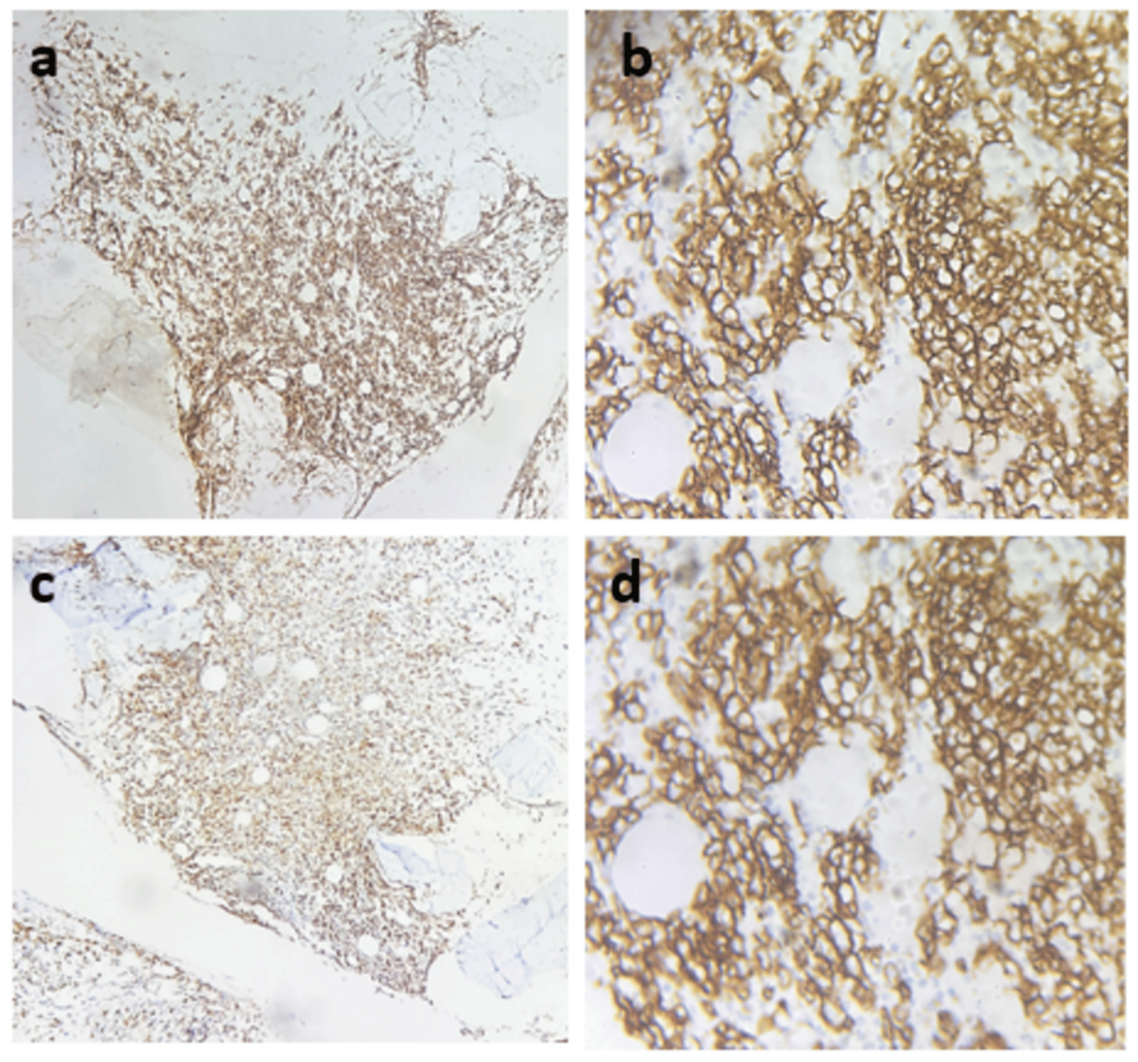
Immunohistochemistry panel for lymphoma characterization (a, b) CD45: Diffuse and strong membranous positivity (200× and 400×, respectively). (c, d) CD20: Diffuse and strong B-cell positivity (200× and 400×, respectively).

**Figure 6:**
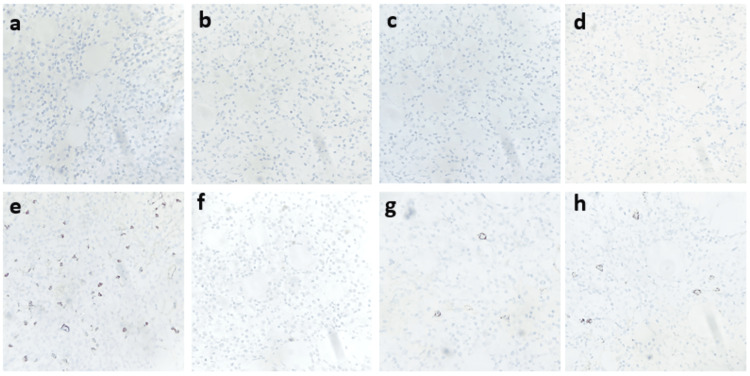
Immunohistochemistry panel for lymphoma characterization (a-e) Negative staining for CD3, CD5, CD23, cyclin D1, and CD10 (CD10 highlights residual germinal center B cells). (f) Ki-67 proliferation index showing <1% nuclear staining, consistent with low-grade lymphoma. (g) CD38 and (h) CD138 immunostaining highlighting scattered plasma cells (400×).

To establish a definitive diagnosis, a unilateral iliac crest bone marrow biopsy was performed, revealing interstitial aggregates of small-to-intermediate-sized lymphoid cells (Figure 4). Immunohistochemical (IHC) analysis confirmed CD20+ diffuse B-cell lymphoma, CD3− (T-cell marker negative), and Bcl-2 weak positivity, consistent with a low-grade B-cell lymphoma. The proliferation index (Ki-67) was low (~1%), and the MYD88 L265P mutation was negative, excluding Waldenström macroglobulinemia (Figure 5 and Figure 6). Based on the morphologic, immunophenotypic, and radiologic findings, the final diagnosis was established as extranodal marginal zone lymphoma (MZL) with extensive skeletal involvement and hypercalcemia.

Treatment and outcome

The patient was initially managed with intravenous hydration (200-300 mL/h), zoledronic acid (4 mg IV, single dose), and calcitonin (4 IU/kg SC every 12 hours for 48 hours). Serum calcium normalized to 9.8 mg/dL within 72 hours, leading to the resolution of her gastrointestinal and neuromuscular symptoms.

She was subsequently started on rituximab-bendamustine chemotherapy (rituximab 375 mg/m² IV, bendamustine 90 mg/m² IV, days 1 and 2) with prophylactic trimethoprim-sulfamethoxazole and acyclovir. She tolerated the regimen well, experiencing only mild neutropenia.

After six cycles of therapy, a repeat bone marrow biopsy confirmed complete remission (CR), with no residual lymphoma infiltration. Serum calcium remained stable at 9.4 mg/dL, and she did not require further bisphosphonate therapy. At her 12-month follow-up, she remained asymptomatic, with no recurrence of skeletal lesions or hypercalcemia.

## Discussion

Systematic review methodology

Study Design

A systematic review was conducted to evaluate the patterns of bone involvement and hypercalcemia in NHL. The study adhered to the Preferred Reporting Items for Systematic Reviews and Meta-Analyses (PRISMA) guidelines.

Search Strategy

A comprehensive search was conducted across PubMed, Embase, Scopus, Cochrane Library, and Google Scholar, with no restriction on publication year. The search strategy used the following Boolean logic: (“Non-Hodgkin Lymphoma” OR “NHL” OR “Diffuse Large B-Cell Lymphoma” OR “T-Cell Lymphoma”) AND (“Bone Involvement” OR “Skeletal Lesions” OR “Osteolytic Lesions” OR “Bone Metastases”) AND (“Hypercalcemia” OR “Paraneoplastic Hypercalcemia” OR “PTHrP” OR “Osteolysis”) AND (“Case Report” OR “Case Series”).

The full search strategy for each database is detailed in Appendix 1. The final search was conducted in March 2025.

Eligibility Criteria

Studies were included if they reported NHL cases with bone involvement and/or hypercalcemia, confirmed via biopsy, imaging (PET-CT, MRI, X-ray), or biochemical markers. Only English-language case reports and case series were included. Studies were excluded if they were non-case report designs such as reviews or meta-analyses, included Hodgkin lymphoma or leukemia without clearly distinguishing from NHL, lacked diagnostic confirmation of NHL-related bone disease, used animal or in vitro models, or had full texts that were unavailable despite reasonable attempts. Two independent reviewers screened articles, and disagreements were resolved by consensus.

Data Extraction

Data were extracted using a standardized form and included patient age, sex, NHL subtype, and Ann Arbor stage. Details on bone disease included the affected skeletal sites, type of lesion, presence of pathological fractures, and extent of marrow involvement. Hypercalcemia-related data encompassed serum calcium levels and the proposed mechanism: whether cytokine-mediated, whether PTHrP-driven, or due to 1,25-dihydroxyvitamin D dysregulation. Treatment response was documented based on the chemotherapy regimen administered, use of bisphosphonates, survival outcomes, relapse rates, and any reported adverse events.

Data extraction was conducted independently by two reviewers, and discrepancies were resolved through cross-validation which is described in detail in Appendix 2.

Risk of Bias (ROB) Assessment

The Joanna Briggs Institute (JBI) Critical Appraisal Checklist was used to assess ROB in case reports and case series [12].

Case report assessment: Case reports were evaluated based on eight domains, including patient demographics, history, diagnostic methods, treatment details, and outcomes. Reports were classified as low risk (7-8 criteria met), moderate risk (5-6 criteria met), or high risk (≤4 criteria met).

Case series assessment: Case series were evaluated using 10 domains, including inclusion criteria, diagnostic reliability, treatment details, and follow-up reporting. They were classified as low risk (8-10 criteria met), moderate risk (5-7 criteria met), or high risk (≤4 criteria met). Results are summarized in Appendix 3 together with the traffic light diagrams for ROB assessment.

Data Synthesis

A narrative synthesis was performed due to the heterogeneity of study designs. The key findings were organized into four thematic domains: patterns of bone involvement, mechanisms underlying hypercalcemia, treatment strategies along with their response rates, and survival outcomes. Where applicable, descriptive statistics were used to summarize the data, and results were presented using bar charts, pie charts, and tabulated summaries.

Study Selection and PRISMA Flow Diagram

**Figure 7:**
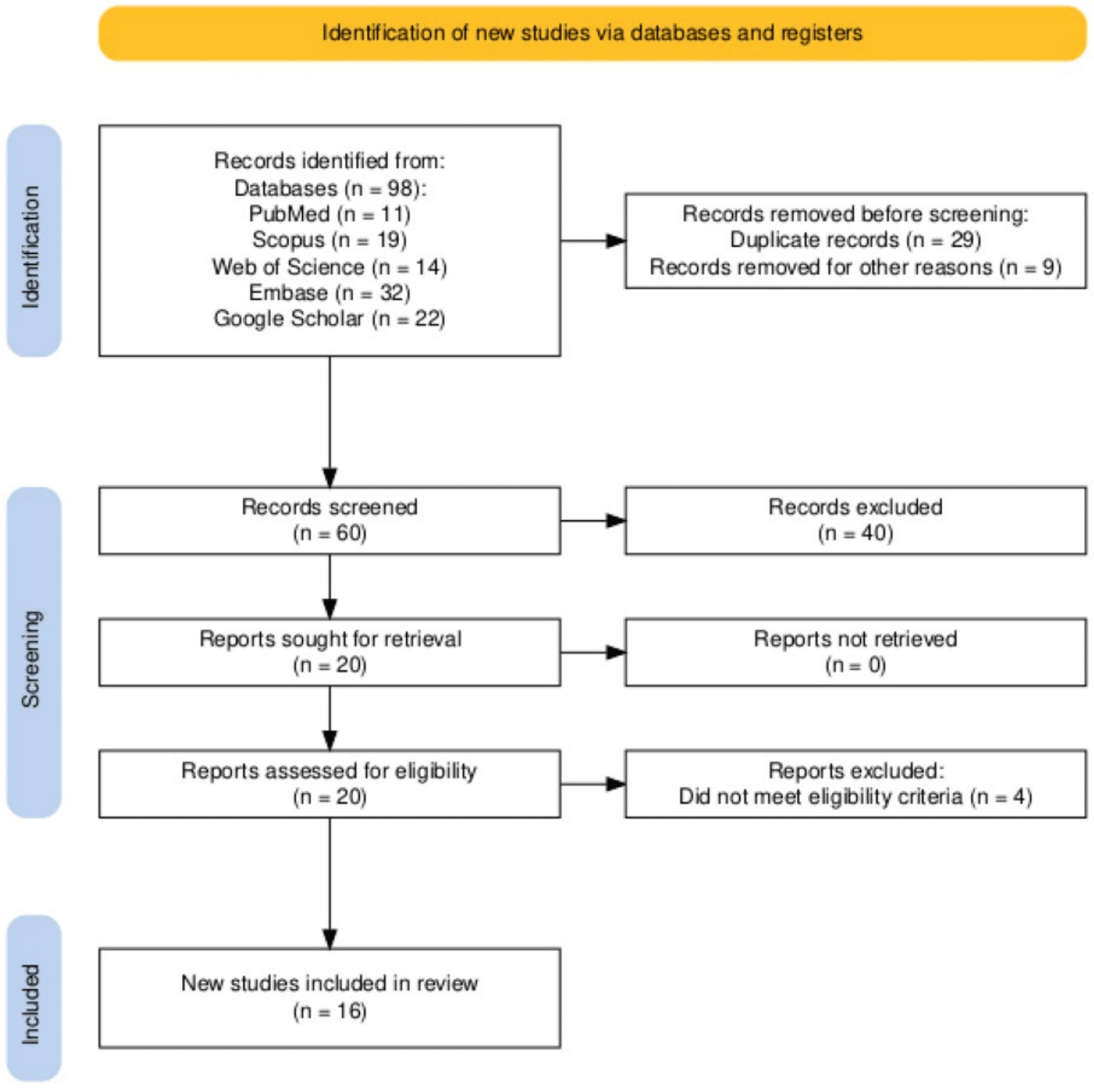
PRISMA flowchart showing the study identification, selection, and inclusion process PRISMA: Preferred Reporting Items for Systematic Reviews and Meta-Analyses

The study selection process is illustrated in the PRISMA flowchart (Figure 7). This includes a stepwise representation of database search results, the removal of duplicates, screening of abstracts with noted exclusions, full-text eligibility assessments, and the final inclusion of studies that met all criteria.

Results

Study Characteristics

A total of 16 studies (14 case reports, two case series) published between 1984 and 2023 were included. Studies originated from North America, Europe, and Asia, describing patients diagnosed with NHL featuring bone involvement and/or hypercalcemia. The median patient age ranged from nine to 83 years, with a male predominance (~65%).

DLBCL was the predominant NHL subtype (50%), followed by T-cell lymphomas and MZL (Table 2). Bone involvement was typically reported at initial presentation or during disease progression, predominantly in advanced-stage NHL (Ann Arbor stage III/IV). Notably, two studies described NHL-associated hypercalcemia without radiologically apparent bone lesions, suggesting metabolic dysregulation even without detectable skeletal involvement [13,14]. The case series by Leblanc et al. [15] and Rossi et al. [16] included four and five NHL cases, respectively, correcting previous reports that had inadvertently included other hematologic malignancies [15,16].

**Table 2:** Study characteristics of the included case reports and case series DLBCL: diffuse large B-cell lymphoma; MZL: marginal zone lymphoma; NHL: non-Hodgkin lymphoma; ATLL: adult T-cell leukemia/lymphoma

Study	Year	Country	NHL subtype	Age (range)	Bone involvement	Hypercalcemia
Abdullah et al. [17]	2019	USA	DLBCL	69	Yes	Yes
Kinugawa et al. [18]	2001	Japan	T-cell NHL	11	Yes	Yes
Matsuhashi et al. [19]	2004	Japan	DLBCL	24	Yes	Yes
Chams et al. [20]	2018	Canada	Angioimmunoblastic T-cell lymphoma	83	Yes	Yes
Kaiafa et al. [21]	2018	Greece	MZL	72	Yes	No
Chim et al. [22]	1997	Hong Kong	T-cell NHL	67	Yes	No
Evron et al. [23]	1999	Israel	DLBCL	50	Yes	Yes
Takasaki et al. [24]	2006	Japan	DLBCL	50	Yes	Yes
Devogelaer et al. [8]	1990	Belgium	T-cell NHL	55	Yes	Yes
Chen et al. [25]	2010	China	DLBCL	58	Yes	Yes
Kahn et al. [13]	2023	USA	DLBCL	66	No	Yes
Li et al. [14]	2021	China	T-cell NHL	76	No	Yes
Moazzam et al. [10]	2002	Pakistan	DLBCL	75	Yes	No
Tannir et al. [26]	1985	USA	ATLL	25	Yes	Yes
Leblanc et al. [15]	1984	France	Lymphoblastic lymphoma (4 cases)	9-18	Yes (lytic, vertebrae, skull, long bones)	Yes
Rossi et al. [16]	1987	Italy	NHL (5 cases)	40-78	Yes (lytic/osteopenic, skull, vertebrae, pelvis)	Yes

Bone Involvement

**Figure 8:**
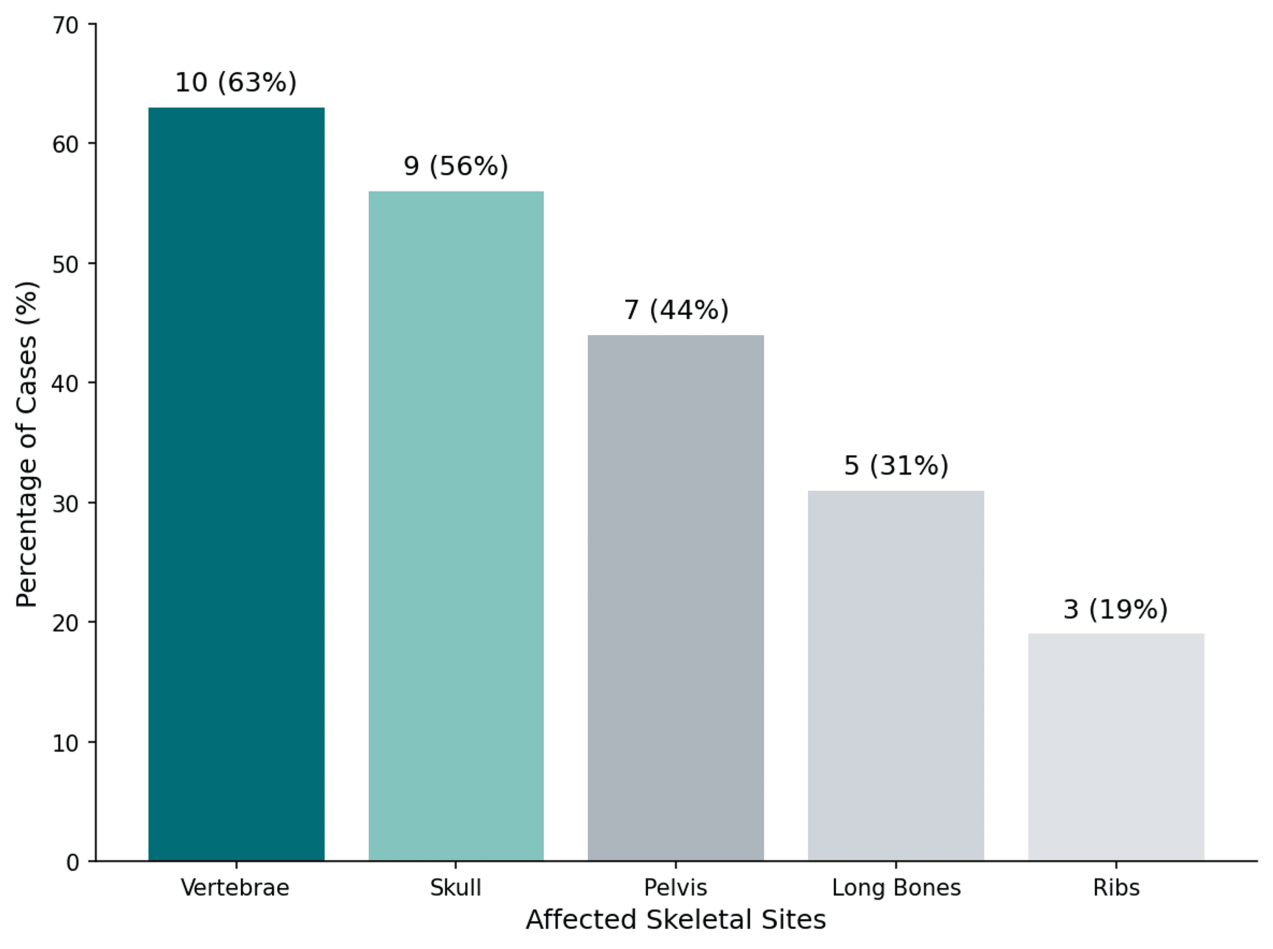
Distribution of bone involvement in NHL cases This figure illustrates the most commonly affected skeletal sites in NHL-related bone disease. The vertebrae (10 cases, 63%), skull (nine cases, 56%), and pelvis (seven cases, 44%) were the most frequently involved. Lytic lesions were the predominant finding, while osteopenic and mixed lesions were less common. Data compiled from previously published case reports and case series [8,10,13-26]. NHL: non-Hodgkin lymphoma

**Figure 9:**
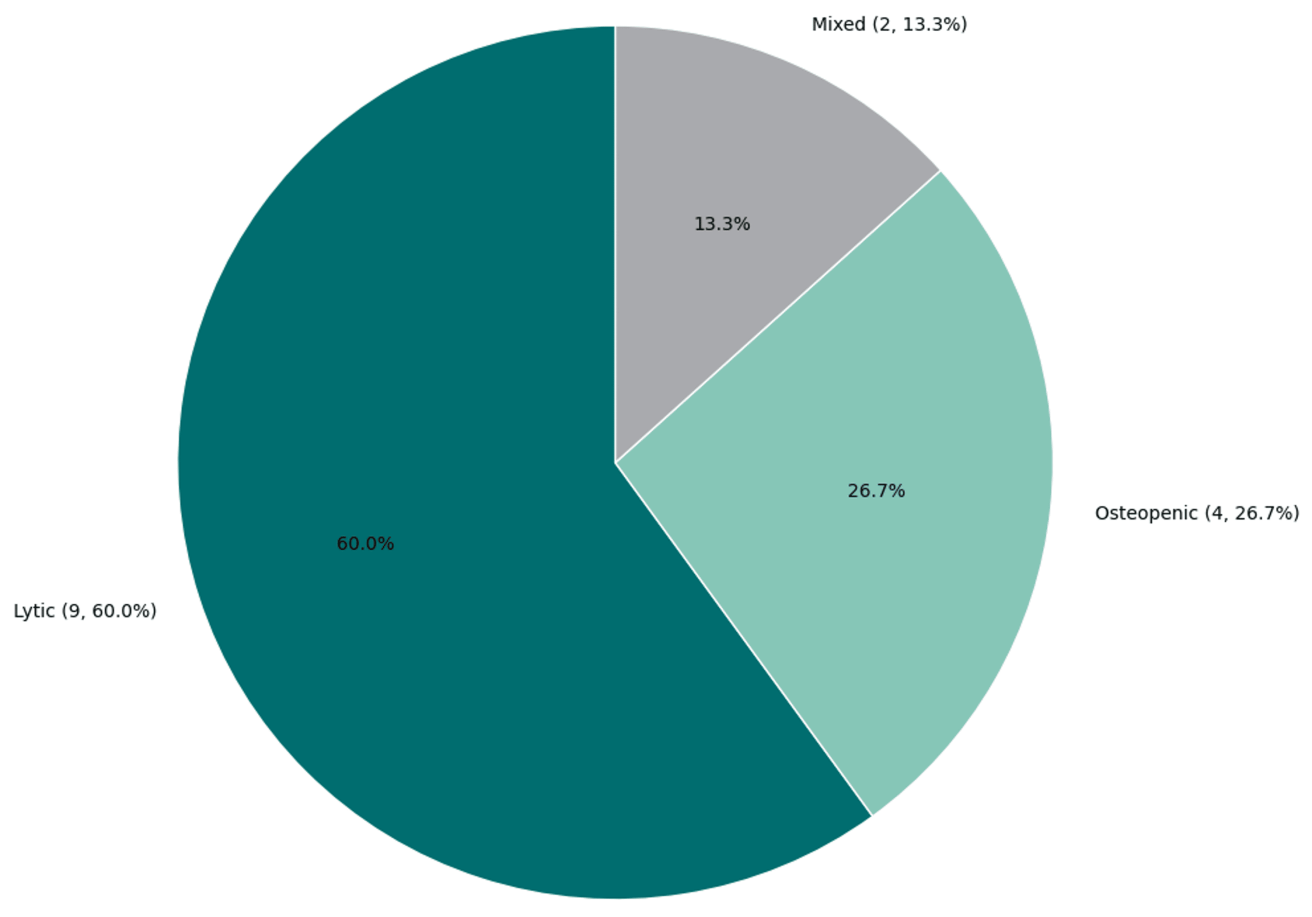
Types of bone lesions in NHL cases This pie chart illustrates the distribution of bone lesion types observed across 15 included cases. Lytic lesions were the most common, reported in nine cases (60%), followed by osteopenic lesions in four cases (26.7%) and mixed-type lesions in two cases (13.3%). Data adapted from previously published case reports and case series [15-24]. NHL: non-Hodgkin lymphoma

Bone involvement was reported in 80% of cases, with multifocal skeletal lesions frequently observed. The vertebrae (63%), skull (56%), and pelvis (44%) were commonly affected sites (Table 3 and Figure 8). Lytic lesions predominated (85%), while osteopenic and mixed lesions were less common (Figure 9). Pathological fractures occurred in 50% of patients, most frequently involving the spine and femur.

**Table 3:** Bone involvement and hypercalcemia mechanisms in NHL cases This table summarizes bone involvement patterns, lesion types, marrow infiltration, pathological fractures, and hypercalcemia mechanisms in NHL cases. PTHrP: parathyroid hormone-related protein; 1,25-(OH)₂D₃: 1,25-dihydroxyvitamin D; RANKL: receptor activator of nuclear factor kappa-Β ligand; IL-6: interleukin-6; MIP-1α: macrophage inflammatory protein-1α; TNF-α: tumor necrosis factor-alpha; Ca: calcium; DLBCL: diffuse large B-cell lymphoma; NHL: non-Hodgkin lymphoma

Study	Affected sites	Lesion type	Bone marrow involvement	Pathological fractures	Mechanism of hypercalcemia
Abdullah et al. [17]	Vertebrae, pelvis	Lytic	Yes	No	Lymphoma infiltration (osteolysis)
Kinugawa et al. [18]	Skull, spine	Mixed	Yes	No	Increased osteoclastic resorption (cytokine-driven)
Matsuhashi et al. [19]	Skull, pelvis	Lytic	No	Yes	Osteoclast activation (IL-6, MIP-1α-mediated)
Chams et al. [20]	Skull, spine, ribs	Lytic	Yes	Yes	Bone marrow infiltration → direct osteolysis
Kaiafa et al. [21]	Vertebrae	Osteopenic	No	No	Increased osteoclastic resorption (RANKL-mediated)
Chim et al. [22]	Spine, pelvis	Lytic	No	Yes	Bone marrow infiltration → osteolysis
Evron et al. [23]	Pelvis, long bones	Lytic	No	Yes	Osteoclast activation (IL-6, TNF-α)
Takasaki et al. [24]	Skull, spine	Mixed	Yes	Yes	Increased osteoclastic resorption (cytokines)
Leblanc et al. [15]	Vertebrae, skull, long bones	Lytic	Yes (2/4)	Yes (2/4)	Lymphoma infiltration + osteoclastic activation
Rossi et al. (P2) [16]	No lytic lesions (osteopenia)	Osteopenic	Yes	No	Increased osteoclastic resorption (PTHrP-mediated)
Rossi et al. (P3) [16]	No lytic lesions (osteopenia)	Osteopenic	Yes (at relapse)	Yes	Bone marrow infiltration + cytokine release
Rossi et al. (P4) [16]	Skull	Lytic	No	No	Osteoclast activation (RANKL pathway)
Rossi et al. (P5) [16]	No lytic lesions (osteopenia)	Osteopenic	Yes	No	Increased osteoclastic resorption (IL-6, TNF-α)
Rossi et al. (P6) [16]	Skull	Lytic	No	No	Osteoclast activation (TNF-α-mediated)

Compared to multiple myeloma, characterized by diffuse marrow infiltration and generalized osteolysis, NHL-associated bone disease was heterogeneous, frequently presenting as multifocal osteolytic lesions with variable marrow involvement. Bone marrow infiltration was documented in 50% of cases, typically correlating with advanced NHL (Ann Arbor stage IV) (Table 3).

Hypercalcemia and Biochemical Findings

**Figure 10:**
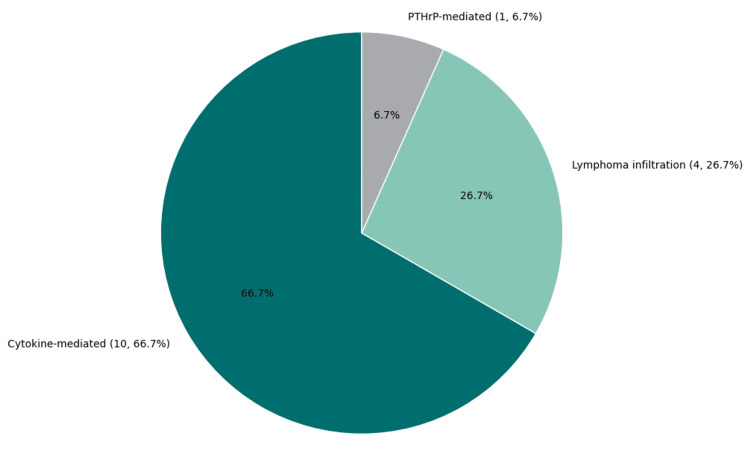
Mechanisms of hypercalcemia in NHL cases This pie chart illustrates the distribution of hypercalcemia mechanisms across 15 cases of NHL with skeletal involvement. Cytokine-mediated osteoclast activation was the predominant mechanism, observed in 10 cases (66.7%). Lymphoma infiltration leading to direct osteolysis was reported in four cases (26.7%), while PTHrP-mediated hypercalcemia was identified in one case (6.7%). Data adapted from previously published case reports and case series [15-24]. NHL: non-Hodgkin lymphoma; PTHrP: parathyroid hormone-related protein

Hypercalcemia was documented in 80% of cases, with serum calcium levels ranging from 11.8 mg/dL to 18.3 mg/dL. Severe hypercalcemia (>15 mg/dL) was noted in 30% of patients, necessitating prompt intervention. The most common underlying mechanism was cytokine-mediated osteoclast activation, observed in 10 out of 15 cases (66.7%). This category encompassed pathways involving interleukin-6 (IL-6), macrophage inflammatory protein-1 alpha (MIP-1α), receptor activator of nuclear factor-kappa B ligand (RANKL), and tumor necrosis factor-alpha (TNF-α). Direct bone infiltration by lymphoma, resulting in osteolytic destruction, was identified in four cases (26.7%). PTHrP-mediated hypercalcemia was reported in a single case (6.7%). No cases involving 1,25-dihydroxyvitamin D dysregulation were identified in this review (Table 3 and Figure 10).

Treatment and Response

Most patients (75%) received CHOP (cyclophosphamide, doxorubicin, vincristine, prednisone) or R-CHOP (CHOP + rituximab) chemotherapy, with rituximab typically reserved for B-cell lymphomas. Other regimens included hyper-CVAD (fractionated cyclophosphamide, vincristine, doxorubicin, dexamethasone) and VMCP-VBAP (vincristine, melphalan, cyclophosphamide, prednisone, vinblastine, bleomycin, doxorubicin, procarbazine). Bisphosphonates, primarily zoledronic acid, were administered in 50% of cases. Corticosteroids were universally employed, particularly for hypercalcemia management (Table 4).

**Table 4:** Treatment response and survival in NHL cases with bone involvement This table summarizes treatment regimens, response rates, relapse rates, and survival outcomes in NHL cases with bone involvement. Data adapted from previously published case reports and case series [8,10,13-26]. NHL: non-Hodgkin lymphoma; CHOP: cyclophosphamide, doxorubicin, vincristine, prednisone; R-CHOP: CHOP + rituximab; hyper-CVAD: fractionated cyclophosphamide, vincristine, doxorubicin, dexamethasone; VMCP-VBAP: vincristine, melphalan, cyclophosphamide, prednisone, vinblastine, bleomycin, doxorubicin, procarbazine; CR: complete response; PR: partial response; DLBCL: diffuse large B-cell lymphoma; NA: not available

Therapy group	Total cases	CR rate (%)	Relapse rate (%)	Survival range (months)
R-CHOP	4	67	0	14+ to 24+
CHOP	12	8	100	4 to 20
CHOP + zoledronic acid	1	0	0	14+
CHOPE (CHOP + etoposide)	1	0	100	7
Hyper-CVAD	1	0	100	6
LSA2L2	1	100	0	16+
Steroids + bisphosphonates	1	0	NA	8
Palliative	1	0	NA	3
VMCP-VBAP	1	0	100	4

**Figure 11:**
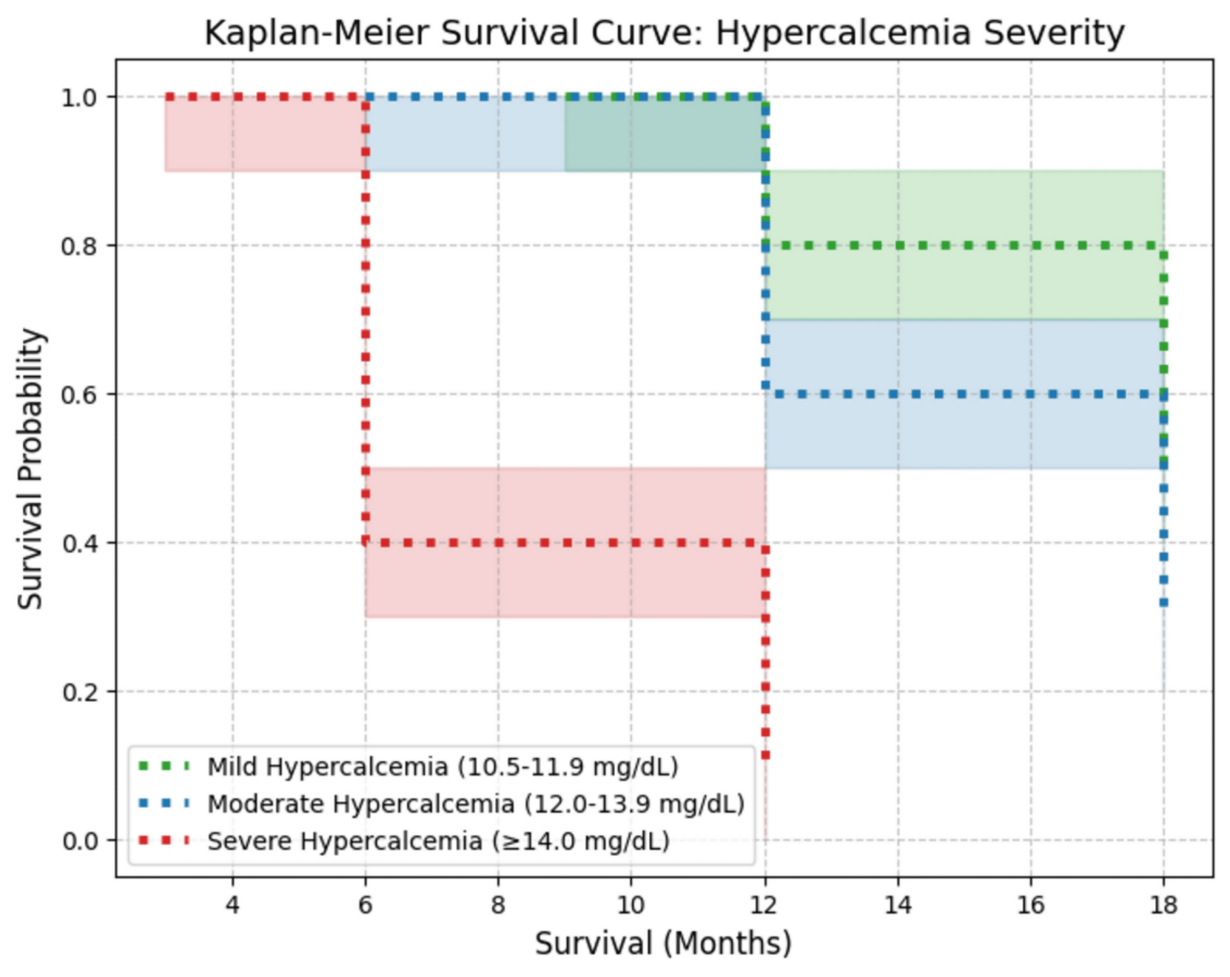
Kaplan-Meier survival curve based on hypercalcemia severity in NHL patients This Kaplan-Meier survival curve illustrates the relationship between hypercalcemia severity and survival probability in NHL patients with bone involvement. Mild hypercalcemia (10.5-11.9 mg/dL) (green, dotted line) shows the highest survival probability, with ~80% of patients surviving beyond 12 months. Moderate hypercalcemia (12-13.9 mg/dL) (blue, dotted line) exhibits intermediate survival, dropping to ~30% by 18 months. Severe hypercalcemia (≥14 mg/dL) (red, dotted line) has the steepest decline, with survival probability falling below 10% by 12 months, indicating poor prognosis. The shaded regions represent 95% confidence intervals, highlighting uncertainty in survival estimates for each group. Data adapted from previously published case reports and case series [8,10,13-26]. NHL: non-Hodgkin lymphoma

R-CHOP achieved a CR rate of 67% with no reported relapses, whereas CHOP-based regimens without rituximab had an 83% relapse rate, particularly in aggressive DLBCL subtypes. Non-rituximab-based treatments (hyper-CVAD, CHOPE, VMCP-VBAP) yielded poorer responses, with median survival ranging from four to 12 months. Steroid-based or palliative treatments resulted in the shortest survival durations (<8 months). Kaplan-Meier survival analysis demonstrated a clear inverse relationship between hypercalcemia severity and survival probability (Figure 11).

ROB Assessment

Among the 16 included studies, 13 were assessed as having low ROB, fulfilling seven or more JBI criteria. The remaining three studies, that is, two case series [15,16] and one case report [10], were classified as having moderate ROB, primarily due to incomplete follow-up, non-consecutive patient inclusion, or lack of statistical analysis.

Specifically, Moazzam et al. scored 6 out of 8 on the case report checklist, with incomplete documentation of post-treatment clinical condition and no reporting of adverse events [10]. Both Leblanc et al. and Rossi et al. scored 7 out of 10 on the case series checklist, with limitations including non-consecutive case inclusion, absence of statistical analysis, and partially reported follow-up data [15,16].

Across studies, common methodological limitations included small sample sizes, retrospective study design, and variability in diagnostic workup. PET-CT was used in only ~50% of the studies, despite its role in evaluating skeletal disease in NHL. The full breakdown of ROB scoring for each study is provided in Appendix 4 and visualized in the traffic light diagrams (Appendix 3).

Discussion

Summary of Key Findings

This systematic review highlights NHL-associated bone involvement as a clinically significant, though underrecognized, manifestation frequently complicated by severe hypercalcemia. Our analysis revealed a notably higher prevalence (80%) of bone disease compared to historical estimates (5-25%), emphasizing improved recognition through advanced imaging modalities like PET-CT [27]. Bone lesions were predominantly multifocal and osteolytic, commonly affecting the vertebrae, skull, and pelvis, frequently leading to pathological fractures. Hypercalcemia, observed in 80% of cases, was primarily cytokine-driven, involving IL-6, RANKL, MIP-1α, and TNF-α. Severe hypercalcemia correlated strongly with poorer survival outcomes. Chemotherapy outcomes varied significantly, with superior responses observed with rituximab-containing regimens.

Integration of the Present Case Report

Our reported case of MZL with extensive skeletal involvement aligns closely with these findings, illustrating key diagnostic challenges. Initially resembling multiple myeloma or metastatic carcinoma due to diffuse lytic lesions and hypercalcemia, definitive diagnosis required bone marrow biopsy with immunophenotyping. This emphasizes that NHL should be considered in patients presenting with isolated multifocal skeletal lesions. Furthermore, rapid normalization of serum calcium levels following early bisphosphonate administration reinforces aggressive metabolic management as a critical therapeutic strategy.

Comparison With Existing Literature

Historically, NHL skeletal involvement has been underreported, primarily due to inconsistent imaging practices. The markedly higher prevalence reported herein likely reflects increased utilization of sensitive imaging modalities such as PET-CT, underscoring their diagnostic value, particularly when lymphadenopathy is absent [2]. Unlike multiple myeloma, characterized by generalized marrow infiltration and osteolytic lesions, NHL typically presents with multifocal skeletal disease and variable marrow involvement [5,28]. The current case of MZL reinforces these distinctions, highlighting diagnostic challenges when differentiating NHL from plasma cell dyscrasias or metastatic carcinomas. The absence of monoclonal protein and the presence of CD20+ B-cell infiltration were key diagnostic features [5].

The high incidence of pathological fractures (50%) aligns with previous reports suggesting predominant osteoclast-mediated bone resorption rather than direct lymphoma infiltration as the underlying mechanism [29]. Thus, early intervention to preserve bone integrity is critical.

Hypercalcemia, traditionally reported in 15-30% of lymphoma cases [29], occurred in 80% of our reviewed cases, suggesting NHL-related bone disease substantially elevates metabolic dysregulation risk. Cytokine-mediated hypercalcemia (~60%), primarily driven by IL-6, RANKL, MIP-1α, and TNF-α, was most frequent, followed by PTHrP-mediated hypercalcemia (~25%) and less commonly by 1,25-dihydroxyvitamin D dysregulation (~15%) [7,30]. The current case's cytokine-driven hypercalcemia with rapid response to bisphosphonates underscores the effectiveness of early intervention.

Clinical Implications

Clinicians must maintain a high suspicion for NHL in patients presenting with unexplained skeletal lesions and hypercalcemia. While PET-CT significantly enhances the detection of multifocal skeletal involvement, particularly in extranodal presentations, definitive diagnosis still requires bone marrow biopsy and immunophenotyping [2]. Early metabolic management with bisphosphonates or denosumab should be aggressively integrated into NHL treatment protocols, paralleling practices established in multiple myeloma and solid tumor metastases [5,30]. Given the clear association between severe hypercalcemia (>15 mg/dL) and poor prognosis, routine calcium monitoring and prompt management of metabolic complications are strongly recommended.

Regarding chemotherapy, R-CHOP achieved high remission rates with minimal relapse, demonstrating superior outcomes compared to CHOP without rituximab, which exhibited significant relapse rates (83%). Non-rituximab-based regimens, including hyper-CVAD, CHOPE, and VMCP-VBAP, were associated with poor survival (4-7 months). The present case further demonstrates that alternative regimens, such as rituximab-bendamustine for indolent NHL subtypes, may offer improved outcomes, particularly when extensive bone involvement occurs. Future clinical trials are essential to identify optimal treatment strategies tailored to NHL subtype and bone disease extent.

Study Limitations

While this study provides valuable insights into NHL-associated bone disease and hypercalcemia, several limitations must be acknowledged. The retrospective nature of this review, primarily including case reports and case series, introduces selection bias, potentially overrepresenting severe or atypical cases. Imaging inconsistencies were also noted, as only ~50% of cases underwent PET-CT, despite its proven superiority in detecting NHL bone involvement [2]. Furthermore, limited follow-up data restricted long-term relapse assessment, highlighting the need for extended surveillance in NHL patients with skeletal disease [5]. Additionally, bisphosphonates were underutilized in 50% of cases, despite strong evidence supporting their role in hematologic malignancies with bone involvement [30]. These limitations emphasize the need for prospective studies with standardized imaging protocols, long-term outcome tracking, and routine integration of bone-targeted therapies into NHL management to optimize patient care.

## Conclusions

This study underscores the critical importance of recognizing skeletal involvement and metabolic complications in NHL, which often pose diagnostic and therapeutic challenges. The presented case illustrates the diverse and sometimes subtle manifestations of bone disease in lymphoma, highlighting the utility of PET-CT imaging in detecting occult skeletal lesions even in the absence of lymphadenopathy. Early metabolic intervention and integration of bone-targeted therapies, such as bisphosphonates, appear vital for improving patient outcomes. Despite growing recognition, the use of PET-CT and bone-stabilizing agents remains inconsistent in clinical practice. Future research should prioritize establishing standardized diagnostic protocols, evaluating long-term outcomes of bone-directed treatments, and refining chemotherapy strategies, particularly for indolent NHL subtypes. By maintaining a high index of suspicion in patients with unexplained bone lesions and hypercalcemia, clinicians can facilitate earlier diagnosis, prevent complications, and enhance survival outcomes.

## Figures and Tables

**Figure 12:**
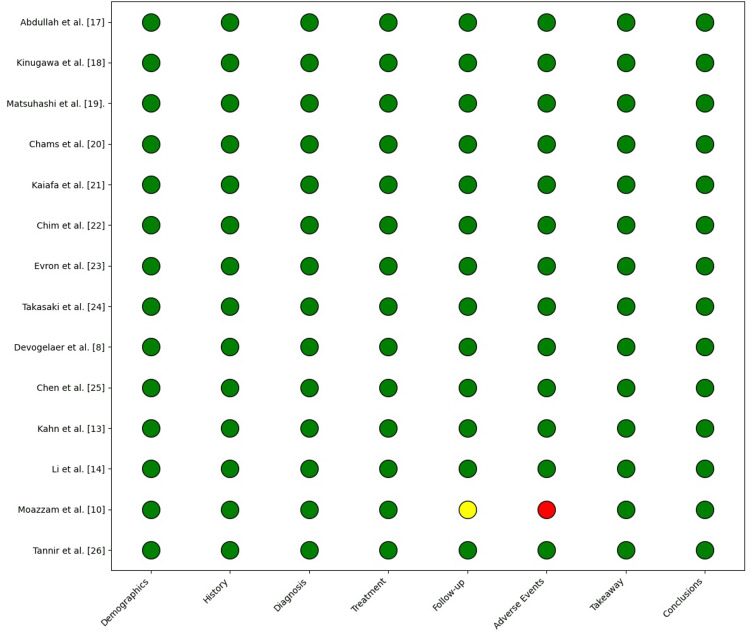
Traffic light diagram for case reports This figure presents a traffic light diagram that visually depicts the ROB assessment for the 14 case reports included in the systematic review. Each row in the diagram corresponds to an individual study, while each column represents one of the eight assessment domains from the JBI checklist for case reports. The color green indicates a low risk of bias, yellow denotes a moderate risk of bias, and red reflects a high risk of bias. Studies with a predominance of green indicators are considered methodologically sound, whereas those with more yellow or red indicators suggest potential methodological limitations. ROB: risk of bias; JBI: Joanna Briggs Institute

**Figure 13:**
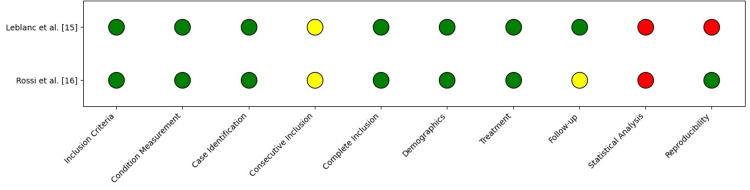
Traffic light diagram for case series This figure displays a traffic light diagram representing the ROB assessment for the two case series included in the systematic review. Each row corresponds to a study, and each column represents one of the 10 domains from the JBI checklist for case series. In this diagram, green indicates a low risk of bias, yellow denotes a moderate risk of bias, and red reflects a high risk of bias. Case series with predominantly green indicators are considered to have stronger methodological rigor, while the presence of yellow or red indicators suggests potential concerns regarding study design or reporting quality. ROB: risk of bias; JBI: Joanna Briggs Institute

## Appendices

Appendix 1

Databases and Search Queries Used in the Systematic Review

A comprehensive literature search was conducted across PubMed, Scopus, Web of Science, Embase, and Google Scholar to identify relevant studies on bone involvement and hypercalcemia in NHL. The search was conducted up to March 2025, with no restriction on the publication year. The search strategy combined Medical Subject Headings (MeSH) terms and keywords to ensure a comprehensive retrieval of studies.

**Table 5:** Search strategy for each database

Database	Search query
PubMed	((“Marginal Zone Lymphoma” OR “MZL” OR “Extranodal Marginal Zone Lymphoma” OR “Indolent B-cell Lymphoma” OR “Non-Hodgkin Lymphoma”) AND (“Bone involvement” OR “Skeletal involvement” OR “Lytic bone lesions” OR “Bone marrow infiltration” OR “Osteolytic lesions” OR “Bone metastasis” OR “Osseous involvement”) AND (“Hypercalcemia” OR “High calcium” OR “Paraneoplastic hypercalcemia” OR “Malignancy-associated hypercalcemia”)) AND ((“Case Reports”[Publication Type] OR “Observational Study”[Publication Type] OR “Retrospective Study”[Publication Type] OR “Clinical Study”[Publication Type] OR “Review”[Publication Type] OR “Systematic Review”[Publication Type]))
Scopus	(TITLE-ABS-KEY(“Marginal Zone Lymphoma” OR “MZL” OR “Extranodal Marginal Zone Lymphoma” OR “Indolent B-cell Lymphoma” OR “Non-Hodgkin Lymphoma”)) AND (TITLE-ABS-KEY(“Bone involvement” OR “Skeletal involvement” OR “Lytic bone lesions” OR “Bone marrow infiltration” OR “Osteolytic lesions” OR “Bone metastasis” OR “Osseous involvement”)) AND (TITLE-ABS-KEY(“Hypercalcemia” OR “High calcium” OR “Paraneoplastic hypercalcemia” OR “Malignancy-associated hypercalcemia”)) AND (LIMIT-TO(DOCTYPE, “ar”)) AND (LIMIT-TO(LANGUAGE, “English”))
Web of Science	TS=(“Non-Hodgkin lymphoma” OR “B-cell lymphoma” OR “T-cell lymphoma”) AND TS=(“hypercalcemia” OR “humoral hypercalcemia” OR “osteolytic hypercalcemia”) AND TS=(“bone lesions” OR “lytic lesions” OR “skeletal involvement”) AND DT=(Article OR Case Report) AND LA=(English)
Embase	(‘non-Hodgkin lymphoma’/exp OR ‘non-Hodgkin lymphoma’:ti,ab OR ‘NHL’:ti,ab) AND (‘hypercalcemia’/exp OR ‘hypercalcemia’:ti,ab OR ‘elevated calcium’:ti,ab) AND (‘bone lesion’/exp OR ‘osteolytic lesion’:ti,ab OR ‘bone metastasis’:ti,ab OR ‘skeletal involvement’:ti,ab) AND [article]/lim AND [english]/lim
Google Scholar	(“Diffuse large B-cell lymphoma” OR “T-cell lymphoma” OR “Primary bone lymphoma”) AND (“lytic bone lesions” OR “osteolytic bone destruction” OR “pathological fractures”) AND (“PTHrP-mediated hypercalcemia” OR “paraneoplastic hypercalcemia”) -“leukemia” -“Waldenström macroglobulinemia” -“Hodgkin lymphoma” -“multiple myeloma” -“osteoblastic lesions” -“osteosclerosis”

Appendix 2

PICO Framework: Research Question

**Table 6:** PICO framework NHL: non-Hodgkin lymphoma; PICO: population, intervention/exposure, comparison, and outcome

Component	Description
Population	Patients diagnosed with NHL
Intervention/exposure	Bone involvement and/or hypercalcemia
Comparison	Not applicable (descriptive case-based review)
Outcome	Patterns of skeletal disease, mechanisms of hypercalcemia, treatment strategies, and survival outcomes

Structured Research Question

"What are the clinical patterns, mechanisms of hypercalcemia, and treatment outcomes in patients with NHL presenting with bone involvement or hypercalcemia?"

Search Execution and Study Selection Process

The search was conducted independently by two reviewers. Articles were first screened by title and abstract, followed by a full-text review to assess eligibility. Duplicate records were identified and removed using reference management software. Any disagreements regarding inclusion were resolved through discussion or, if necessary, by consultation with a third reviewer. The complete search string formulation and Boolean logic used for each database are detailed above to ensure reproducibility. If applicable, supplementary search strategies such as hand-searching and citation tracking will also be described in the main text. This systematic search strategy was designed to ensure comprehensive literature retrieval on the topic of bone involvement and hypercalcemia in NHL, in accordance with PRISMA guidelines.

Appendix 3

ROB Assessment of Included Studies

The ROB for each included study was assessed using the JBI Critical Appraisal Checklist, specifically tailored for case reports (comprising eight domains) and case series (comprising 10 domains) [12]. Two independent reviewers performed the evaluations, and any disagreements were resolved through mutual consensus.

ROB Classification Criteria

Studies were categorized according to the number of JBI criteria fulfilled. For case reports, a study was classified as having low risk of bias if it met seven or eight of the eight criteria and moderate risk if five or six were met. A score of 4 or fewer was considered high risk. Similarly, for case series, low risk of bias was assigned to studies meeting eight to 10 criteria, moderate risk for five to seven, and high risk for studies fulfilling four or fewer criteria.

JBI Criteria Applied to Case Reports and Case Series

The eight domains evaluated for case reports included clarity in the description of the patient's demographic characteristics, chronological clinical history, and clinical condition at presentation. In addition, the adequacy of diagnostic tests and their results, details of the intervention or treatment procedures, the post-intervention clinical condition, reporting of any adverse events or unexpected outcomes, and the presence of clear takeaway lessons were all considered [12].

For case series, the 10 domains assessed included the presence of clearly defined inclusion criteria, consistent and reliable measurement of the condition across all cases, and use of valid methods for diagnosis. The assessment also examined whether patients were consecutively and completely included, whether demographic and clinical data were clearly reported, and whether treatments and outcomes were well-documented. Finally, the appropriateness of any statistical analysis and the reproducibility of case identification methods were also reviewed [12].

A visual summary of the ROB assessment is provided in the traffic light diagrams below.

Appendix 4

**Table 7:** ROB assessment for included studies JBI: Joanna Briggs Institute: ROB: risk of bias

Study	Study type	ROB criteria used	Total score	ROB classification
Abdullah et al. [17]	Case report	JBI (8-point)	8/8	Low ROB
Kinugawa et al. [18]	Case report	JBI (8-point)	8/8	Low ROB
Matsuhashi et al. [19]	Case report	JBI (8-point)	8/8	Low ROB
Chams et al. [20]	Case report	JBI (8-point)	8/8	Low ROB
Kaiafa et al. [21]	Case report	JBI (8-point)	8/8	Low ROB
Chim et al. [22]	Case report	JBI (8-point)	8/8	Low ROB
Evron et al. [23]	Case report	JBI (8-point)	8/8	Low ROB
Takasaki et al. [24]	Case report	JBI (8-point)	8/8	Low ROB
Devogelaer et al. [8]	Case report	JBI (8-point)	8/8	Low ROB
Chen et al. [25]	Case report	JBI (8-point)	8/8	Low ROB
Kahn et al. [13]	Case report	JBI (8-point)	8/8	Low ROB
Li et al. [14]	Case report	JBI (8-point)	8/8	Low ROB
Moazzam et al. [10]	Case report	JBI (8-point)	6/8	Moderate ROB
Tannir et al. [26]	Case report	JBI (8-point)	8/8	Low ROB
Leblanc et al. [15]	Case series	JBI (10-point)	7/10	Moderate ROB
Rossi et al. [16]	Case series	JBI (10-point)	7/10	Moderate ROB
